# Evaluation of gastrointestinal symptoms in different patient groups using the visual analogue scale for irritable bowel syndrome (VAS-IBS)

**DOI:** 10.1186/1471-230X-11-122

**Published:** 2011-11-10

**Authors:** Mariette Bengtsson, Oskar Hammar, Thomas Mandl, Bodil Ohlsson

**Affiliations:** 1 Department of Nursing, Faculty of Health and Society, Malmö University, Sweden; 2 Department of Clinical Sciences, Gastroenterology Division, Skåne University Hospital, Malmö, Lund University, Lund, Sweden; 3 Department of Clinical Sciences, Rheumatology Division, Skåne University Hospital, Malmö, Lund University, Lund, Sweden

## Abstract

**Background:**

Irritable bowel syndrome (IBS) and gastrointestinal (GI) dysmotility disorders have a similar clinical picture, although dysmotility disorders require the attention of a specialist. Patients with primary Sjögren's syndrome (pSS) have also been described to suffer from IBS-like symptoms. No objective marker is available to distinguish between the patients. A visual analogue scale has been developed for IBS patients (VAS-IBS) to measure treatment response of GI symptoms and well-being in patients with IBS. The aim of the present study was to examine if VAS-IBS could be used to compare the degree of GI complaints in different patient populations, to get an objective marker to differentiate between the patients.

**Methods:**

The VAS-IBS consists of 7 VAS scales, namely, abdominal pain, diarrhoea, constipation, bloating and flatulence, vomiting and nausea, psychological well-being and the intestinal symptoms' influence on daily life. Consecutive female patients suffering from IBS, dysmotility disorders and pSS were asked to complete the VAS-IBS questionnaire when visiting the out-patient clinics. In addition, a control population consisting of healthy female volunteers was included.

**Results:**

Healthy volunteers had almost no GI symptoms, whereas all 3 patient groups expressed symptoms. There was no statistical significant difference between IBS and dysmotility in any of the scales besides vomiting and nausea (p = 0.044). Except for constipation, patients with pSS had less severe symptoms than the others.

**Conclusion:**

The VAS-IBS questionnaire could be used to assess the level of GI symptoms. However, VAS scores do not help the clinicians to differentiate between IBS and other dysmotility disturbances.

## Background

Irritable bowel syndrome (IBS) is a common, functional, gastrointestinal (GI) disorder, affecting a significant number of people, predominantly women [1]. Its aetiology and pathophysiology is insufficiently understood. Abdominal pain and bloating are the dominant and most troublesome symptoms of IBS [1]. It is difficult in clinical practice to estimate the symptomatic changes occurring in patients with IBS based on their description. It is equally difficult in description and to evaluate the effect of different treatments. Hence, there is a need to translate the patients' perception of their symptoms and their subjective well-being into quantitative parameters. Several disease-specific questionnaires have been developed during the last years to try to evaluate GI symptoms in an objective manner. However, most of these are too time-consuming to be used in daily clinical practice. The visual analogue scale for IBS (VAS-IBS) has therefore been developed and validated, offering a short and patient-reported questionnaire to be used in clinical practice for these patients [2]. The original purpose to develop this questionnaire was to measure treatment response of GI symptoms and well-being in patients with IBS.

Primary Sjögren's syndrome (pSS) is an autoimmune disease mainly affecting exocrine glands, leading to decreased secretion and mucosal dryness. Recently, we have described that a great number of these patients also suffer from functional bowel symptoms [3,4]. Enteric dysmotility (ED) and chronic intestinal pseudo-obstruction (CIPO) are GI diseases presenting with the same type of symptoms as functional bowel diseases, but with objective signs of dysmotility disturbances and/or histopathological changes in the bowel wall [5,6]. Clinically, it may be difficult to differentiate between severe IBS and dysmotility disorders at the initial meeting. The latter are patients who need more advanced health care than IBS patients and should be cared for by specialists in gastroenterology [7,8]. Sometimes patients with dysmotility disorders are not correctly diagnosed for many years [7]. No objective markers are easily available to differentiate between the entities at first contact. Besides sharing the same type of symptoms with unclear aetiology, patients with both IBS, CIPO, ED and pSS have in common expression of high serum titres of antibodies against gonadotropin-releasing hormone (GnRH) [3,9-11]. The hypothesis was that the VAS-IBS scale could be an objective marker to distinguish between these patient populations, thereby helping the clinicians to tailor a correct examination and treatment.

The aim of the present study was thus to compare the degree of GI complaints in different patient populations known for their presence of functional bowel symptoms and high prevalences of GnRH antibodies, to get objective markers for differentiation between patients at an early stage, and to see if any group of patients with symptoms of dysmotility could be simply identified using the VAS-IBS scale.

## Methods

This study was performed according to the Helsinki declaration and was approved by the Ethics Committee of Lund University. Patients were consecutive patients at Skåne University Hospital, Malmö. All patients and controls gave informed consent before entering the study. As symptoms of functional bowel disorders may differ between men and women, and the vast majority of these patients are women, only women were studied.

### Subjects

#### Controls

The control group was recruited among hospital staff and consisted of 52 healthy female volunteers (median age 44 years, range 22 - 77 years) who had not undergone prior abdominal surgery.

#### Patients with irritable bowel syndrome (IBS)

All consecutive female patients visiting the out-patient clinic at the Department of Gastroenterology, during a 2-year period, suffering from abdominal pain and symptoms of altered bowel habits were investigated according to standard norms depending on the severity of symptoms. Investigation comprising radiological and/or endoscopic examination to rule out organic disease was performed when clinically indicated. Appropriate laboratory samples were analyzed. Patients with GI symptoms but without abnormal findings on the examinations mentioned above, who fulfilled the Rome-III criteria, were classified as IBS [1]. These 39 patients (median age 37 years, range 18-69 years) were thus invited to participate in this study. None of the patients declined to participate. All patients were working full time, and were not dependent on nutritional support or opioid analgesics. However, their psychological state was not examined. They were all referred back to the primary health care center from which they came for continued care after examination at the University Hospital.

#### Patients with dysmotility disorders

Consecutive female patients subjected to laparoscopic full-thickness biopsy at the Department of Surgery or Gastroenterology, during a 10-year period, because of severe GI pain and dysmotility were included. In addition, patients with symptoms of severe dysmotility who underwent intestinal resection within the same time frame were included, but were not considered for laparoscopic biopsy because full-thickness bowel wall tissue was already available for histochemical examination and thereby histopathological classification [9,10]. They all underwent extensive examinations with oesophagogastroduodenoscopy, enterography or capsule endoscopy, ileocolonoscopy and abdominal ultrasound depending on the symptoms present. In some cases this had already been performed repeatedly for several years. Routine analyses were performed on blood and urinary samples. The GI examination was then further completed with oesophageal manometry, gastric emptying scintigraphy, antroduodenojejunal manometry, colonic transit time and full-thickness biopsies of the distal ileum, depending on the clinical picture [5,6,9,10,12-14].

Patients who fulfilled the 3 criteria: a medical history compatible with pseudo-obstruction, documented events or chronic signs mimicking mechanical obstruction (bowel dilatation and/or air/fluid levels) and absence of mechanical obstruction or other organic cause for these symptoms and findings were classified as CIPO [6]. The criteria for ED were gastrointestinal symptoms that were severe enough to warrant referral for small bowel manometry, presence of abnormal contractile activity, but absence of sub-occlusion episodes and absence of any medication that could lead to observed motor abnormalities [5]. These patients represent the vast majority of cases of suspected CIPO/ED in the southern part of Sweden, as the listed departments constitute the tertiary referral centre for GI dysmotility in this region.

Thirty-one female patients were recognised in a retrospective manner. Twenty-one patients (median age 43 years, range 26-84 years) accepted to participate in the study. Out of these, 16 had ED and 5 had CIPO. As a previously study had shown that there was no significant difference in symptoms between these 2 groups, they were regarded as one group in the statistical calculations [7,8]. Ten of the patients had peroral nutrition, whereas 11 had supplements of enteral or intravenous nutrition. Seventeen of the patients used opioid analgesics. Only 4 of the patients worked full time, and 2 and 1 worked 75% and 50% of full time, respectively. Ten were retired because of disease and 1 because of age.

#### Patients with primary Sjögren's syndrome (pSS)

Thirty-five consecutive female patients with pSS at the Department of Rheumatology, according to the American-European Consensus Criteria (AECC) [15], who had previously been included in a study on autonomic dysfunction (AD) [16], were asked to participate in this study. Twenty-six pSS patients (median age 62 years, range 29-65 years) were willing to be included in the study. None of the patients had previously undergone GI surgery.

### Questionnaire

#### Self estimation on gastrointestinal symptoms - VAS-IBS questionnaire

Patients estimated 7 different entities on a VAS scale from 0 - 100 mm where 0 represents very severe problems and 100 represents absence of problems. The 7 entities were abdominal pain, diarrhoea, constipation, bloating and flatulence, vomiting and nausea, perception of mental well-being and the intestinal symptoms' effect on daily life. This questionnaire has formerly been developed and psychometrically tested for patients with GI symptoms without organic reasons [2].

The questionnaire was sent by mail to all participants with dysmotility disorders and all controls, and returned to the investigator in an envelope, after completion. Patients with IBS and pSS completed the questionnaire at the visit at the departments.

### Statistical analyses

Values are given as median, interquartile range (IQR). As the pSS patient group was significantly older than the other groups, variables were age-standardized using a linear regression model into which age was added as a covariate and the variables were expressed as z-scores. When comparing VAS scores between groups, the age-standardized values were used. Differences between groups were calculated by Kruskal-Wallis test. There were statistically significant differences between groups, why the calculations were followed by Mann Whitney U test, which are the values shown. P < 0.05 was considered statistically significant.

## Results

### Controls

The control group did not differ in age compared to patients with IBS and dysmotility (data not shown), but were younger than the pSS patients (p = 0.000). Age did not significantly influence the variables in the VAS-IBS (data not shown). Healthy subjects scored high values on the VAS scale (median values 95-100, interquartile ranges 78-100), except for bloating and flatulence that was present also in controls (86 (71-99)).

### Irritable bowel syndrome (IBS) and dysmotility disorders

Both patients with IBS and dysmotility disorders rated their GI symptoms as more severe compared to controls. Both groups differed significantly from controls in all variables. There was no statistical difference in any of the individual symptoms between these two groups, except for vomiting and nausea (Table 1). Influence of GI symptoms on daily life was rated as the worst symptom, followed by bloating and flatulence. Although they reported great impact on daily life, their overall psychological well-being was not affected to the same extent (Table 1).

**Table 1:** Comparison between the groups - evaluation of their symptoms being estimated with visual analogue scale for irritable bowel syndrome (VAS-IBS).

VAS-IBS score	Controls = 52	IBS = 39	Dysmotility = 21	pSS = 26	Statistically significant p values
Abdominal pain					
Z-score	0.22 (-0.41-0.60)	-3.43 (-4.23 - -2.77)	-3.34 (-4.42 - -2.31)27 (12-44) ***	-1.08 (-2.31 - 0.04)	IBS vs pSS p = 0.000
Absolute score	95 (85-99)	23 (10-37) ***		73 (54-97) ***	pSS vs Dysm p = 0.000

Diarrhoea					
Z-score	0.27 (-0.11-0.55)	-3.91 (-5.23 - -0.53)	-3.23 (-4.11 - -0.55)	-0.75 (-1.97-0.15)	IBS vs pSS p = 0.012
Absolute score	97 (90-100)	35 (19-83) ***	49 (33-69) ***	82 (66-98) ***	pSS vs Dysm p = 0.049

Constipation					
Z-score	0.32 (-0.39-0.76)	-0.69 (-2.99-0.54)	-1.57 (-3.32-0.60)	0.03 (-2.41-0.63)	
Absolute score	91 (78-99)	72 (30-95) **	54 (18-95) *	86 (42-97) *	

Bloating, flatulence					
Z-score	0.28 (-0.26-0.77)	-2.33 (-2.84 - -1.06)	-2.15 (-2.67 - -1.28)	-0.62 (-1.61-0.31)	IBS vs pSS p = 0.000
Absolute score	86 (71-99)	17 (3-50) ***	18 (7-45) ***	63 (37-87) ***	pSS vs Dysm p = 0.002

Vomiting, nausea					
Z-score	0.27 (0.08-0.35)	-3.34 (-4.55 - -0.03)	-4.82 (-4.81 - -0.78)	0.08 (-3.14-0.20)	IBS vs pSS p = 0.004 IBS vs Dysm p = 0.044
Absolute score	98 (97-100)	50 (34-94) ***	32 (12-84) ***	97 (54-99) ***	pSS vs Dysm p = 0.001

Psychological well-being					
Z-score	0.51 (-0.09-0.70)	-2.08 (-3.29 - -0.56)	-1.79 (-1.79-0.46)	-0.76 (-2.29-0.62)	IBS vs pSS p = 0.018
Absolute score	96 (84-100)	45 (23-74) ***	51 (22-94) **	72 (44-98) ***	

Influence on daily life					
Z-score	0.53 (-0.12-0.59)	-3.05 (-3.43 - -2.49)	-3.06 (-3.06 - -2.62)	0.20 (-1.75-0.49)	IBS vs pSS p = 0.000
Absolute score	98 (82-100)	11 (3-25) ***	8 (3-22) ***	88 (42-96) ***	pSS vs Dysm p = 0.000

All p values are calculated using the z-scores. Mann Whitney U test. Patients vs controls; * < 0.05, ** < 0.01, *** < 0.001. IBS = Irritable bowel syndrome, pSS = primary Sjögren's syndrome.

### Primary Sjögren's syndrome (pSS)

All variables differed significantly between controls and patients with pSS (Table 1). Patients with pSS rated their GI symptoms as less severe than patients with IBS and dysmotility. They had significantly less severe symptoms than IBS patients in all variables except for constipation (p = 0.186). Compared to patients with dysmotility disorders, they differed in all variables except constipation (p = 0.247) and psychological well-being (p = 0.252) (Table 1).

## Discussion

In this study, we estimated GI symptoms in 3 different patient cohorts with known GI complaints without gross organic changes in the bowel wall. The results showed that patients with pSS had less severe GI complaints than patients with primary GI diagnosis such as IBS and dysmotility disorders, which did not differ in symptoms except for vomiting and nausea.

Surprisingly, there was not any difference in any parameter measured in our study between patients with IBS and dysmotility disorders except for vomiting and nausea. Patients with dysmotility disorders have pathological examinations when examining GI motility and histopathological changes in full-thickness biopsies [5,6]. They are often dependent on nutritional support to keep weight and avoid malnutrition, are dependent of analgesics and are not able to work [7]. On the contrary, patients with IBS have no objective changes pathognomic for the disease. Instead, the diagnosis is set when patients fulfil the Rome III criteria, based on presence of various GI symptoms [1]. Most IBS patients maintain normal weight and are able to eat without nutritional support, work full-time and never use opioid analgesics [17]. Patients with dysmotility disorders are considered to have a severe chronic GI disease which needs advanced care by specialists [7,8], whereas patients with IBS according to recommendations should be managed in the primary health care system, or by themselves [17]. Still, IBS patients, in the present study, score their GI symptoms as severe as patients with dysmotility disorders do.

Our results are in contrast to another study showing that IBS patients have less severe GI symptoms and better quality of life than CIPO patients [18]. It has been shown that significant impairments of health-related quality of life can only be detected in IBS patients with severe symptoms [19]. Female IBS patients have more severe GI symptoms and a reduced quality of life compared to male patients, and female patients at a hospital have reduced quality of life compared to patients at a primary care centre [20]. Hence, our patients constitute a highly selected group. Some explanations to the different results in our study compared to Cogliandro et al. [18] may depend on our smaller sample size, and that patients of both genders were included in the latter study, whereas only women were included in the present study. Both studies were performed at a tertiary GI centre, why selected IBS patients with severe symptoms were included. Thus, our results may not be applied to the general IBS population in the community, but may reflect the IBS patients often seen at a specialist centre. If this study on IBS patients had been performed at a primary health care centre, there had maybe been a difference in the VAS scales compared to patients with dysmotility disorders. Further, our patients estimated their symptoms on a continuous VAS scale, whereas the patients in the study by Cogliandro et al. [18] scored their symptoms 0-4, which could also affect the results.

Patients with IBS may have varying degrees of the disease. Those who seek care are considered sicker than those who do not, with a higher proportion of abnormal personality patterns, greater illness behaviour and lower life events scores than non-patients with IBS [1]. Patients with IBS are associated with an enhanced perception of personal vulnerability to illness and over-reporting of symptoms [21,22]. This may be due to the fact that a high percentage of IBS patients suffer from somatization disorders [23], which in turn may lead to over-reporting of GI symptoms, wide-spread pain, migraine, psychiatric symptoms, as well as in an increase of physicians consulted, medication changes, poor treatment outcomes and treatment dissatisfaction compared to their counterparts without somatization disorders [24,25]. Half of the IBS patients have been shown to suffer from anxiety and/or depression in several studies [25,26]. Depression and anxiety scores were significantly correlated with VAS ratings of perceived pain and overall discomfort [27]. When examining the neuronal responses in the brain by functional magnetic resonance imaging (fMRI), there were significant differences in the neural processing of pain between IBS patients and controls, supporting the role of affective disturbances in the neural processing of visceral pain in IBS, and further underlining the importance of psychological factors in the pathophysiology of visceral hyperalgesia in these patients [28,29]. The results in the present study which showed that the IBS patients experienced their GI symptoms as severe as patients with dysmotility disorders, might be explained by psychological mechanisms [30,31], as dysmotility patients suffering from severe disease making them unable to eat and work, still scores at levels comparable to those of patients with IBS.

The classical symptoms in patients with pSS are dry eyes and mouth. Recently, we have described that a great number of these patients also suffer from functional bowel symptoms, dysphagia and impaired gastric emptying rate [3,4,32]. Thus, pSS is a disease affecting more than the exocrine glands. It is difficult to say if pSS patients suffer from true functional bowel disorders, or if their symptoms are IBS-like symptoms associated with mucosal inflammation as in the case of inflammatory bowel disease [33], or both. Examinations have shown oesophageal dysmotility and impaired gastric emptying rate in these patients [4,32], whereas the rest of the GI tract has been poorly investigated. They may suffer from dysmotility also in the small and large intestines, explaining their symptoms. On the other hand, signs of gastro paresis were found to be poorly correlated with symptoms of IBS [4]. However, they fulfilled the symptom criteria for IBS and FD in 46% and 89% of cases [3], respectively, and their prevalence of IBS-like symptoms are thus higher than in the general population which is around 10-15% [34,35].

Besides psychological mechanisms, there is growing evidence for involvement of immunological mechanisms in the aetiology of IBS. An increased number of mast cells have been described in the mucosa of patients with IBS [36]. Others have described an increased number of T cells and macrophages [37,38], as well as a correlation between symptom severity and levels of pro-inflammatory cytokines TNFα and IL-1β suggestive of a direct effect of cytokines on visceral sensation [39]. Recently, antibodies against GnRH in serum has been described in patients suffering from functional and dysmotility disorders, but not in patients suffering from coelic disease and inflammatory bowel diseases [3,9-11]. Irritable bowel syndrome thus seems to be a heterogenous disorder involving both peripheral immunological, psychological and central mechanisms. Immunological mechanisms seem to be more dominant in post-infectious IBS, and may be a separate entity of the disease [37-39]. However, also in these patients an over-reporting of symptoms and perception of illness seems to be important [22,23], to explain our results in the present study.

The present results underline the conflict seen in the daily practice. Physicians consider changes that can be objectified as more severe, and treat these patients at specialist departments, whereas patients with functional diagnosis without objective, measurable changes are considered less sick and are treated at primary health care centres. The results of the present study show that the symptoms are as troublesome for the patients, independent of presence of objective changes. The patients need confirmation of their symptoms, and education to handle their symptoms, as shown previously [40].

There are several limitations in this study. First, one limitation of this study is that we have examined patients during treatment. If we had examined patients with dysmotility disorders without nutritional support and analgesics, they had maybe scored worse than the IBS patients. On the other hand, also the IBS patients had some sort of palliative treatment. Secondly, the relatively small patient groups as well as the lack of a population-based control group are a limitation. Finally, the selection of female patients at a tertiary GI centre is also a limitation, making the results hard to apply on the general IBS population in the community.

## Conclusions

Previous studies have shown that the VAS-IBS questionnaire could be used to estimate GI symptoms, and the degree of symptoms to aid in treatment choice, and evaluating possible treatment effects. The present study showed that female IBS patients with functional complaints at a tertiary GI centre experience their symptoms as severe as patients with dysmotility disorders expressing objective, measurable changes on GI examinations. VAS scores do not help the clinician at a tertiary GI centre to differentiate between IBS and more severe dysmotility disturbances. As our patients comprise a selected group, our results may not be applied to the general IBS population in the community.

## Competing interests

The authors declare that they have no competing interests.

## Authors' contributions

All authors together designed the study. OH and BO collected the data from the Departments of Gastroenterology and Surgery, and TM collected the data from the Department of Rheumatology. MB and TM contributed to the statistical analyses. BO wrote the manuscript and financially supported the study (Development Foundations of Region Skane). All authors contributed to the manuscript with constructive criticism, and read and approved the final manuscript.

## Pre-publication history

The pre-publication history for this paper can be accessed here:

http://www.biomedcentral.com/1471-230X/11/122/prepub
